# Laparoscopic and robotic extravascular stenting of the left renal vein for anterior nutcracker syndrome in a single-center series

**DOI:** 10.1016/j.jvsv.2026.102459

**Published:** 2026-02-12

**Authors:** Shuai Tang, Kai Li, Fan Chang, Song Li, Zheng Lv, Jianghui Zhang, Wensong Wu, Huiyuan Shi, Fangmin Chen

**Affiliations:** a Department of Urology, Tianjin Third Central Hospital, Tianjin, China; b College of Medicine, Nankai University, Tianjin, China; c Department of Urology, Wuhan Sixth Hospital, Wuhan, China

**Keywords:** Nutcracker syndrome, Extravascular stenting, Standardization, Fibrotic ring, Operative checklist

## Abstract

**Background:**

Nutcracker syndrome (NCS) arises from extrinsic compression of the left renal vein (LRV) between the superior mesenteric artery and the abdominal aorta. Extravascular stenting (EVS) has emerged as a minimally invasive alternative to historical operations and endovascular stents. We report a single-center series spanning 2010 to 2025 and propose a standardized, reproducible framework that couples intraoperative process quality with objective postoperative hemodynamic targets.

**Methods:**

We retrospectively analyzed 22 consecutive NCS patients treated with laparoscopic or robot-assisted EVS. We standardized five intraoperative steps (five-in-a-row: fibrotic-ring resection, proper length tailoring/placement, sufficient superior mesenteric artery mobilization, complete division of LRV tributaries, stable anterior fixation). Postoperative duplex ultrasound metrics included aortomesenteric (AM) LRV peak systolic velocity and the AM/hilum peak systolic velocity (PSV) ratio. Thresholds were determined by receiver operating characteristic-Youden index; performance was summarized at fixed cutoffs, with bootstrap for the ratio and exploratory OR/AND combinations.

**Results:**

Complete success was achieved in 18 of 22 patients (81.8%). Data-driven analysis identified a postoperative AM PSV of ≤72 cm/s as the primary attainment threshold, yielding a sensitivity of 1.00, specificity of 0.75, accuracy of 0.95, and area under the receiver operating characteristic curve (AUC) of ≈0.917. The AM/hilum ratio showed a Youden-optimal cutoff of ≈1.90 (clinically ≈2.0) with an AUC of ≈0.56, supporting its role as a sensitivity/replicability metric rather than a standalone gatekeeper. OR and AND combinations demonstrated expected trade-offs; a simple 0/1/2 composite score achieved an AUC of ≈0.78. The five-in-a-row checklist was concordant with attaining the AM-PSV target on Doppler ultrasound examination.

**Conclusions:**

Laparoscopic or robot-assisted EVS is a safe, feasible, and effective option for NCS. We a propose postoperative AM PSV of ≤72 cm/s as a unified, reproducible primary quantitative end point, with an AM/hilum ratio of ≈2.0 as a secondary, replicability-oriented metric. Integrating these targets with a standardized five-in-a-row checklist establishes a process-outcome loop that enhances procedural reproducibility and supports sustained symptom relief over the available follow-up.

**Article Highlights**

• **Type of Research:** Single-center retrospective cohort study
• **Key Findings:** In 22 patients with anterior nutcracker syndrome treated with laparoscopic or robot-assisted extravascular left renal vein stenting, complete success occurred in 18 (81.8%) with one self-limited lymphatic leak and one stent migration. A postoperative aortomesenteric peak systolic velocity of ≤72 cm/s predicted complete success (area under the receiver operating characteristic curve, 0.917; sensitivity, 1.00; specificity, 0.75).
• **Take Home Message:** Extravascular left renal vein stenting for anterior nutcracker syndrome can be standardized with a reproducible intraoperative checklist, and a postoperative aortomesenteric peak systolic velocity of ≤72 cm/s is a practical hemodynamic attainment target strongly associated with complete success.

Nutcracker syndrome (NCS) is a rare vascular disorder resulting from compression of the left renal vein (LRV) between the abdominal aorta (AA) and the superior mesenteric artery (SMA), or between the AA and the spine.^1^ Based on the position of the LRV, NCS is classified into anterior (type I) and posterior (type II) types.^2^ Anterior NCS refers to compression of the LRV between the AA and the SMA, representing the most common variant. Posterior NCS, in contrast, involves an anomalous anatomical course of the LRV, typically where it passes between the AA and the spine, resulting in compression.^3^ Clinical manifestations of NCS include hematuria, proteinuria, left flank pain, and left-sided varicocele in males.^4^ Female patients may present with pelvic congestion syndrome (dysmenorrhea, deep dyspareunia, and postcoital pain) owing to elevated LRV pressure.^5^ However, diagnosis is often challenging and delayed. Once NCS is diagnosed, therapeutic intervention should be determined, because long-term LRV hypertension may lead to LRV thrombosis and chronic kidney disease.^6^

The management principles for NCS primarily depend on symptom severity.^7^ There is also a view that conservative treatment can be adopted for patients with a body mass index of <18.5.^8^ Surgical intervention may be necessary for patients with severe symptoms. Surgical options for NCS include open LRV transposition, left renal autotransplantation, nephropexy, left gonadal vein ligation, or left gonadal vein bypass.^9^ However, these procedures are used less frequently owing to the risk of complications such as paralytic ileus, hemorrhage, and thrombosis.^1^ Minimally invasive techniques include endovascular stenting and laparoscopic extravascular stenting (LEVS). Although endovascular stenting is an attractive option, rare complications such as fibromuscular hyperplasia, in-stent restenosis, or migration may necessitate reintervention and require long-term anticoagulation.^10^ Given that LRV compression is extrinsic, caused by the SMA or AA, EVS can effectively relieve LRV stenosis. Compared with other surgical approaches, it offers the advantage of eliminating the need for postoperative antiplatelet therapy and carries a lower risk of complications.^11^

Our group has previously reported experience with laparoscopic EVS (LEVS) for NCS.^12^ Our short-term and midterm outcomes demonstrated the feasibility and safety of this technique. The primary objective of this study was to evaluate the safety and clinical effectiveness of laparoscopic or robot-assisted EVS (RA-EVS) of the LRV for anterior NCS in a consecutive single-center series. Secondary objectives were to (1) define a reproducible, stepwise intraoperative framework (five-in-a-row) to operationalize procedural quality and (2) identify postoperative duplex ultrasound hemodynamic targets associated with complete success. Outcomes were also summarized by surgical approach (laparoscopic vs robot assisted) as an exploratory, descriptive comparison.

## Methods

### Patients

Patients diagnosed with NCS were consecutively enrolled. The inclusion criteria were (1) age between 16 and 60 years, (2) diagnostic imaging findings demonstrating the beak sign jet phenomenon, high-velocity flow in the aortomesenteric (AM) portion with proximal dilation, an LRV peak velocity ratio ≥3, and an LRV diameter ratio ≥3, and (3) presence of persistent hematuria, proteinuria, and intolerable flank/abdominal pain. In male patients, varicocele could be present or absent. The exclusion criteria were (1) coexisting severe underlying diseases or congenital disorders, (2) pregnancy, lactation, or planned pregnancy within 6 months, (3) renal insufficiency or contraindication to contrast-enhanced computed tomography (CT) scans, (4) requirement for long-term oral medication during the study period, and (5) severe psychiatric symptoms precluding cooperation. From March 2010 to March 2025, a total of 22 NCS patients underwent laparoscopic or RA-EVS. The treatment of all patients included in this study was approved by the institutional review board and conformed to the principles of the Declaration of Helsinki.

### Preoperative assessment

Clinical signs and symptoms are essential components in making the diagnosis of NCS. Urine and blood tests were used to examine patients with a suspected history and clinical symptoms, such as recurrent gross hematuria and intermittent left kidney pain without hypertension. Regular cystoscopy and ureteroscopy may reveal that there are no tumors or stones, just blood pouring from the left ureteric orifice. Urinary cytology and excretory urography were used to exclude malignant tumors, stones, or other causes of hematuria. Doppler ultrasound (DUS) was used for assessments of LRV diameter (hilar and AM portion), LRV peak velocity (AM and hilar portion) at the supine position, and other abnormalities such as left varicocele, urinary tract stones. CT, CT angiography, or magnetic resonance angiography was used to demonstrate the anatomy of the LRV and the LRV constrained by the SMA and the AA.

### Surgical technique

#### Ports and positioning

**Fig 1:**
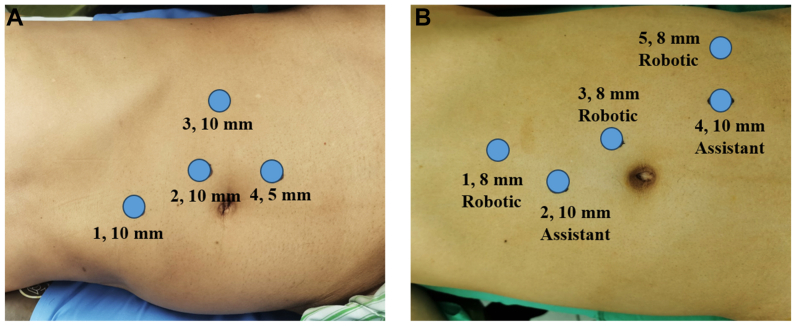
Patient position and port placement. **(A)** Patient position and port placement for laparoscopic extravascular stenting (EVS). **(B)** Patient position and port placement for robot-assisted (RA)-EVS.

For LEVS, patients were placed in a right lateral decubitus position (45°-60°) under general anesthesia. Standard sterile preparation and draping were performed. The procedure was pursued with a standard four-port laparoscopic transperitoneal technique. The first port is located at the midpoint of the xiphoid process to the umbilical line, and a 10-mm trocar is inserted. The second port is 3 cm above the umbilicus and 3 cm apart (10-mm trocar, as the camera port), which is used to establish pneumoperitoneum. The CO_2_ pressure was maintained at a level of about 14 to 15 mm Hg. The third 10-mm trocar port was inserted into the midclavicular line at the subcostal margin at the umbilical level. The fourth 5-mm trocar port is 5 cm below the second port (Fig 1, *A*). The patient's head is lowered 10° to 15° before starting surgery.

For robotic-assisted EVS, the patient was placed in a right lateral decubitus position (45°-60°) after general anesthesia and secured to the operating table. Standard sterile preparation and draping were performed. The first port was located at the midpoint of the xiphoid process to the umbilical line, and an 8-mm Da Vinci robot trocar was inserted. The second port was an assistant port with a 10-mm trocar about 6 cm away from the first and third ports. The third port was 3 cm above the umbilicus and 3 cm apart (8-mm Da Vinci robot trocar, as the camera port). The fourth port was also an assistant port with a 10-mm trocar and was located at the intersection of the midclavicular line and the anterior superior iliac spine, about 6 cm away from the third and fifth ports. The fifth port was an 8-mm Da Vinci robot trocar and was located at the intersection of the anterior superior iliac spine and the anterior axillary line (Fig 1, *B*). The patient's head was lowered 10° to 15°. The pneumoperitoneum CO_2_ pressure was maintained at 14 to 15 mm Hg during the procedure.

The choice between LEVS and RA-EVS was not randomized and reflected routine clinical practice. Approach selection was based on robotic platform availability, surgeon preference, and patient-specific anatomical considerations. To improve transparency regarding potential selection bias, baseline characteristics and outcomes are summarized by approach as descriptive/exploratory comparisons. The same operative objectives and the five-step intraoperative checklist (five-in-a-row) were applied to both approaches.

#### Exposure of LRV and the resection of the fibrous ring

**Fig 2:**
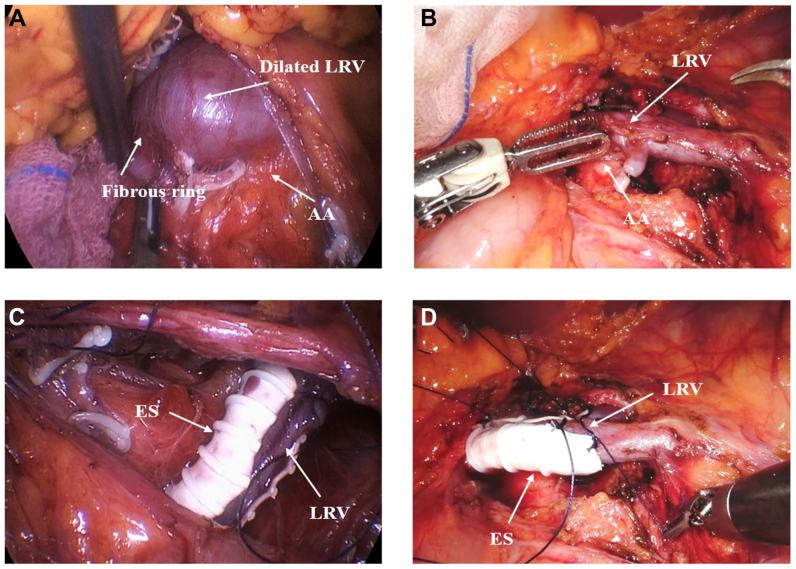
Intraoperative imaging in laparoscopic or robot-assisted extravascular stenting (RA-EVS). **(A)** Exposed dilated left renal vein (*LRV*) and fibrous ring in laparoscopic EVS (*LEVS*). **(B)** Exposed dilated LRV in RA-EVS. **(C)** The extravascular stent was placed around the LRV and sutured in LEVS. **(D)** The extravascular stent was placed around the LRV and sutured in RA-EVS. *AA*, abdominal aorta.

The posterior peritoneum was incised through Toldt's fascial space along the splenic flexure of the left half colon. Then Gerota's fascia was opened at the renal pedicle to expose the LRV. The anterior and posterior walls of the LRV were then completely freed till the entrance of the inferior vena cava (IVC). The LRV, IVC, SMA, and AA were subsequently exposed to reach the IVC, where the LRV appeared with a narrowing segment under pressure between the AA and the SMA. In our group of 22 patients, we discovered a narrow fibrous ring around the LRV outflow adjacent to the IVC in 19 cases (86.4%). The fibrous ring was resected, and the dilated proximal LRV collapsed immediately (Fig 2, *A* and *B*).

#### Placement of an extravascular stent

The length of the compressed AM segment of the LRV was measured to guide extravascular stent planning. All patients received a 12-mm polytetrafluoroethylene (PTFE) cuff. The cuff length was tailored to cover the entire compressed segment with adequate proximal and distal margins. An externally reinforced 12-mm extended PTFE (ePTFE) vascular graft (Bard Peripheral Vascular) was prepared accordingly and cut into a C shape. Then, the graft was wrapped around the LRV to form an extravascular stent to avoid pressure. When the position of the extravascular stent was properly attached, the stent was sutured in front of the AA (right lateral decubitus position) to avoid displacement (Fig 2, *C* and *D*).

#### Dissection of the branches of the renal vein

**Fig 3:**
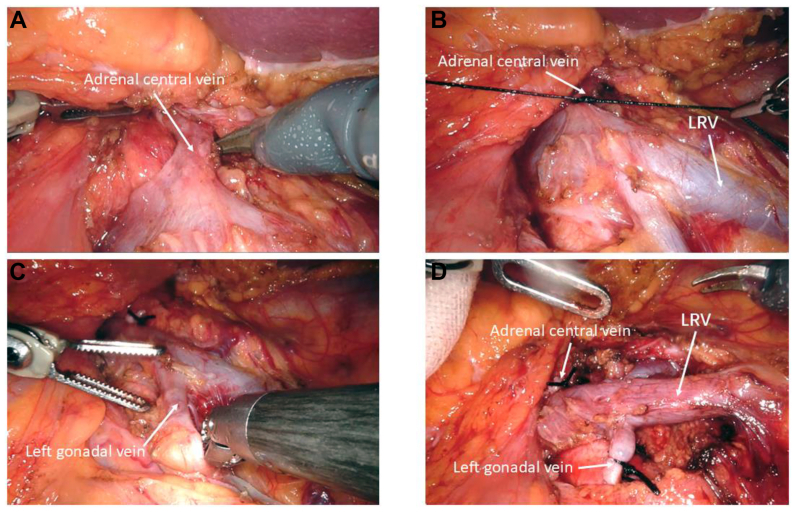
Dissection of the branches of the renal vein. **(A)** Fully expose the left adrenal central vein. **(B)** Ligate the left central adrenal vein. **(C)** Search for the left gonadal vein. **(D)** The left adrenal central vein and left gonadal vein after ligation, as well as the lumbar veins (if any) should also be ligated. *LRV*, left renal vein.

During dissection of the LRV, tributaries such as the central adrenal vein, lumbar vein, or gonadal vein were commonly observed draining into the LRV. For instance, the left central adrenal vein (Fig 3, *A*) typically drains directly into the LRV and must be ligated and dissected (Fig 3, *B*). A similar procedure was conducted for other branches draining into the LRV, such as the lumbar vein and the left gonadal vein (Fig 3, *C*). Intraoperatively, these vessels required individual ligation and dissection (Fig 3, *D*). The purpose of this step was to decrease venous return and thereby alleviate renal congestion. This specific procedure was not performed in patients enrolled in the early phase. Consequently, differences were observed in the outcomes, as detailed in the Results.

### Evaluation and follow-up

Patients were followed for a period ranging from 3 to 40 months postoperatively (average, 23.5 months; interquartile range, 18-29 months). Follow-up was defined as the interval from the index operation to the most recent clinical contact. Routine postoperative follow-up included a standardized 3-month assessment with both duplex ultrasound examination and CT imaging. Duplex ultrasound parameters used for hemodynamic analyses in this study were obtained at this 3-month visit. Beyond 3 months, cross-sectional imaging with a CT scan was not mandatory and was obtained selectively based on clinical indications or patient preference, given that routine long-term CT scans are not standard practice and may impose additional cost and radiation exposure. Subsequent follow-up primarily focused on symptom assessment and urinalysis (to screen for recurrent hematuria or proteinuria), with ultrasound examination performed as clinically indicated.

In this study, the success criteria for laparoscopic or RA-EVS were categorized into symptomatic success and imaging success. Symptomatic success was defined as complete resolution of NCS-related symptoms or return to baseline without clinically meaningful residual symptoms. Imaging success was defined as patent blood flow, reduced vessel diameter, absence of dilation or compression, and no evidence of stent migration or thrombosis. Complete success was defined as the achievement of both imaging success and symptomatic success. Recurrence was defined as a reappearance of NCS-related symptoms after initial symptomatic success.

### Statistical analysis

Data collected for analysis included sex, age, clinical symptoms; preoperative and postoperative laboratory findings (hematuria and proteinuria); preoperative and postoperative imaging characteristics including peak velocity of the LRV at the AM portion, peak velocity of the LRV at the renal hilum, AM angle between the SMA and AA, diameter of the LRV at the renal hilum, diameter of the LRV at the AM portion; and postoperative outcomes of complications or symptom relief. Paired *t* tests were used to assess the significance of differences within groups. A *P* value of <.05 was considered statistically significant. All statistical analyses were performed using SPSS 27.0 software. Additional receiver operating characteristic (ROC) analyses, bootstrap resampling, and logistic regression were performed in Python 3.11. Baseline characteristics and outcomes were summarized stratified by surgical approach (LEVS vs RA-EVS) as descriptive comparisons. Given the small sample size, particularly in the RA-EVS subgroup, formal adjustment methods such as propensity score matching were not performed to avoid unstable estimates and substantial loss of information. Baseline balance between approaches was assessed using absolute standardized mean differences.

Threshold determination and feasibility were evaluated as follows. First, ROC curves were plotted for continuous variables, and the cutoff was chosen at the point that maximized the Youden index J = Sensitivity + Specificity − 1. At this cutoff, we reported sensitivity, specificity, accuracy, positive predictive value, and negative predictive value. Second, to quantify uncertainty in our small sample, we performed nonparametric bootstrap resampling (4000 iterations) on the postoperative ratio to obtain 95% confidence intervals for the optimal cutoff, its associated performance metrics, and the area under the ROC curve (AUC). In parallel, we fitted a logistic regression model to verify the monotonic association that a lower ratio corresponds with a higher probability of complete success, and we plotted the predicted probability-ratio curve. Exploratorily, we compared OR/AND rule-based combinations of the thresholds (AM peak systolic velocity [PSV] ≤ cutoff and ratio ≤ cutoff) for their overall discriminative performance, but these were not used as primary end points. Finally, the main threshold was used to define postoperative attainment, and, together with the process attainment of the five-in-a-row intraoperative checklist, to jointly evaluate the procedure's reproducibility and clinical effectiveness from both process and outcome perspectives.

This retrospective study was approved by the Research Review Committee of Tianjin Third Central Hospital (IRB No. 1,882,023-009-02). Clinical trial registration was not required. The study adhered to ICH-GCP, the Declaration of Helsinki, Good Practice for the Quality Management of Pharmaceutical Clinical Trials, and all applicable local regulations. Written informed consent was obtained from all included patients.

## Results

A total of 22 NCS patients met the inclusion criteria and underwent laparoscopic or RA extravascular LRV stenting. The cumulative preoperative characteristics of these patients are summarized individually in Table I. The mean age at surgery was 28.9 years (range, 16-47 years). The preoperative clinical presentations included gross hematuria in all 22 patients (100.0%), proteinuria in 5 patients (22.7%), anemia in 1 patient (4.5%), flank/abdominal pain in 5 patients (22.7%), and left-sided varicocele in 6 patients (27.3%). Of the 22 patients, 17 underwent LEVS and 5 underwent RA-EVS. Preoperative baseline characteristics are summarized by approach in Table II, with baseline balance further illustrated using absolute standardized mean differences in Supplementary Fig 1 (online only). After surgical intervention, complete success (both symptomatic and imaging success) was achieved in 18 patients (81.8%). Imaging success was attained in three patients (13.6%), but all experienced symptom recurrence. One patient (4.5%) developed stent migration; however, symptomatic success was achieved in this case.

**Table I:** Individual patient preoperative characteristics and treatment details (n = 22)

Patient	Sex	Age	Primary symptoms	AM angle	LRV anteroposterior diameter, mm, and hilum/AMportion (supine)	LRV peak velocity, cm/s, and AM/hilumportion (supine)	Follows-up	Outcomes
1	Male	17	Gross hematuria; varicocele	26	9.2/1.8	106/20	Symptomatic success	Relief
2	Female	31	Gross hematuria	23	8.0/1.2	107/18	Stent migration	Relief
3	Male	16	Gross hematuria; varicocele	14	9.7/1.4	112/21	Symptomatic success	Relief
4	Female	26	Gross hematuria; proteinuria	19	11.9/1.9	98/16	Imaging success	Recurrent proteinuria
5	Male	31	Gross hematuria; varicocele	28	15.2/2.5	115/35	Symptomatic success	Relief
6	Female	28	Gross hematuria; anemia	31	10.8/1.8	110/36	Symptomatic success	Relief
7	Male	40	Gross hematuria; flank pain	18	9.7/1.6	126/34	Symptomatic success	Relief
8	Male	38	Gross hematuria	22	8.8/1.2	124/25	Symptomatic success	Relief
9	Male	34	Gross hematuria; flank pain	26	8.3/2.0	132/30	Symptomatic success	Relief
10	Male	23	Gross hematuria; varicocele	20	8.5/2.1	104/20	Symptomatic success	Relief
11	Male	32	Gross hematuria	22	9.3/1.9	112/18	Imaging success	Recurrent gross hematuria
12	Female	47	Gross hematuria; flank pain	24	8.9/1.7	120/17	Symptomatic success	Relief
13	Male	27	Gross hematuria; varicocele	21	9.2/2.2	107/19	Imaging success	Recurrent gross hematuria
14	Male	30	Gross hematuria	31	10.2/2.3	108/18	Symptomatic success	Relief
15	Female	23	Gross hematuria	22	8.5/1.9	122/23	Symptomatic success	Relief
16	Male	35	Gross hematuria	25	9.2/2.3	117/15	Symptomatic success	Relief
17	Male	20	Gross hematuria; varicocele	21	9.7/2.2	119/22	Symptomatic success	Relief
18	Female	36	Gross hematuria; proteinuria	32	8.8/2.6	101/30	Symptomatic success	Relief
19	Male	17	Gross hematuria; proteinuria; flank pain	26	7.1/2.5	95/31	Symptomatic success	Relief
20	Female	35	Gross hematuria; proteinuria	25	10.6/2.3	98/26	Symptomatic success	Relief
21	Female	33	Gross hematuria; proteinuria	18	10.2/3.1	103/29	Symptomatic success	Relief
22	Female	16	Gross hematuria; flank pain	21	6.5/2.0	95/11	Symptomatic success	Relief

*AM,* Aortomesenteric; *LRV,* left renal vein.

**Table II:** Baseline characteristics by surgical approach (preoperative)

Characteristic	Overall (n = 22)	LEVS (n = 17)	RA-EVS (n = 5)	Abs SMD
Demographics				
Female sex	9 (40.9)	6 (35.3)	3 (60.0)	.49
Symptoms (preoperative)				
Gross hematuria	22 (100.0)	17 (100.0)	5 (100.0)	.00
Microscopic hematuria	22 (100.0)	17 (100.0)	5 (100.0)	.00
Proteinuria	5 (22.7)	3 (17.6)	2 (40.0)	.49
Flank pain	5 (22.7)	4 (23.5)	1 (20.0)	.09
Varicocele	6 (27.3)	4 (23.5)	2 (40.0)	.35
Imaging and duplex ultrasound				
Age, years	30.5 [23.0-34.8]	31.0 [23.0-35.0]	26.0 [16.0-31.0]	.60
SMA angle, degrees	22.5 [21.0-26.0]	23.0 [21.0-26.0]	21.0 [19.0-28.0]	.17
LRV diameter at hilum, mm	9.2 [8.6-10.1]	9.2 [8.5-9.7]	9.7 [8.8-11.9]	.72
LRV diameter at AM portion, mm	2.0 [1.8-2.3]	2.0 [1.8-2.3]	2.0 [1.9-2.5]	.10
Diameter ratio (hilum/AM)	4.5 [4.1-5.8]	4.4 [4.2-5.1]	6.1 [3.4-6.3]	.40
PSV at AM portion, cm/s	109.0 [103.2-118.5]	110.0 [106.0-120.0]	101.0 [98.0-112.0]	.81
PSV at hilum, cm/s	21.5 [18.0-28.2]	22.0 [18.0-29.0]	21.0 [16.0-22.0]	.37
PSV ratio (AM/hilum)	5.3 [3.9-6.0]	5.3 [3.8-5.9]	5.3 [4.6-6.1]	.34

*Abs SMD,* Absolute standardized mean difference; *AM,* aortomesenteric; *LEVS,* laparoscopic extravascular stenting; *LRV,* left renal vein; *PSV,* peak systolic velocity; *RA-EVS,* robot-assisted extravascular stenting; *SMA,* superior mesenteric artery.

Values are number (%) or median [interquartile range].

**Fig 4:**
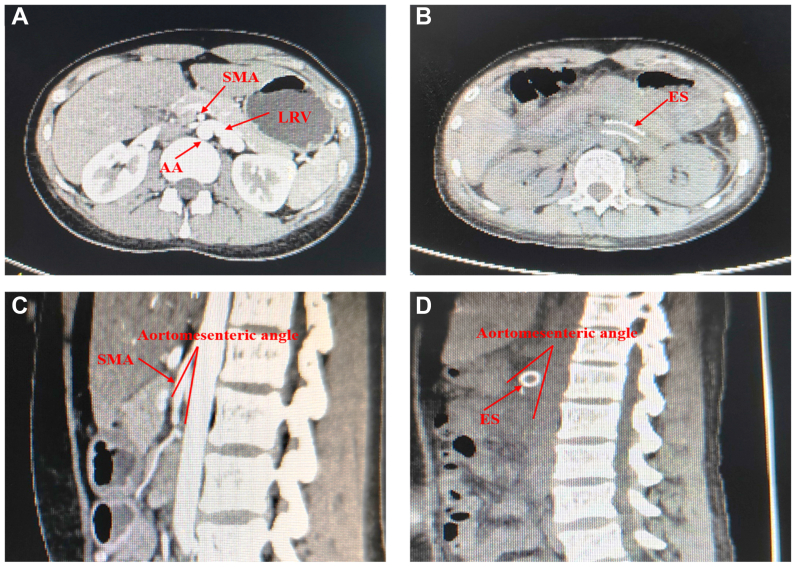
Preoperative and postoperative computed tomography (CT) views of nutcracker syndrome (NCS). **(A) (C)** Preoperative compression of the left renal vein (*LRV*) between the abdominal aorta (*AA*) and the superior mesenteric artery (*SMA*). **(B) (D)** Postoperative placement of the extravascular stent (*ES*).

The CT scan images preoperatively and at 3 months are shown for side-by-side comparison (Fig 4). A representative 1-year CT scan is provided in Supplementary Fig 2 (online only). All procedures were completed without conversion to open surgery. The perioperative parameters for patients with complete success are summarized in Table III. Briefly, significant hemodynamic improvements were evidenced by a marked decrease in the mean LRV peak velocity at the AM portion and a significant decrease in the mean velocity ratio. Anatomically, the AM angle increased and the LRV diameter decreased significantly, resulting in a reduced diameter ratio. All NCS symptoms were gradually improved during follow-up, with abdominal/flank pain and gross hematuria resolving immediately postoperatively. Only one complication occurred (a self-resolving lymphatic leakage after 1 week of drainage).

**Table III:** Comparison of preoperative and postoperative parameters of patients who achieved complete success (n = 18)

Variables	Preoperative	Postoperative	*P* value
LRV peak velocity at AM portion, cm/s	111.5 (95-132)	54.1 (38-70)	<.001
LRV peak velocity at hilar portion, cm/s	24.2 (11-36)	29.4 (20-44)	.057
Peak velocity ratio of AM portion/hilar portion	5.0 (3.1-8.6)	1.9 (1.4-2.3)	<.001
Angle between the SMA and AA	24 (14-31)	38 (27-49)	<.001
LRV diameter at hilar portion, mm	9.5 (6.5-15.2)	6.5 (4.6-9.6)	<.001
LRV diameter at AM portion, mm	2.1 (1.4-3.1)	3.2 (1.9-4.5)	<.001
Diameter ratio of hilar portion/AM portion	4.8 (3.1-7.3)	2.1 (1.3-3.6)	<.001

*AA,* Abdominal aorta; *AM,* aortomesenteric; *LRV,* left renal vein; *SMA,* superior mesenteric artery.

Postoperative duplex measurements were obtained at 3 months after surgery. Data are presented as median (range).

Notably, four patients achieved only symptomatic success or imaging success postoperatively. A comparison of their preoperative and postoperative parameters is presented in Table IV, aiming to identify contributing factors for the limited success. For patient 2, despite the stent being slightly displaced postoperatively, symptomatic success was eventually achieved. The clinical improvements induced decreased LRV diameter ratio and peak velocity ratio, as well as increased SMA-AA angle. Importantly, intraoperative complete ligation and division of all LRV tributaries (central adrenal vein, gonadal vein, and lumbar vein) were performed, a key procedure used in the complete success cases. Although stent migration precluded imaging success, the significant improvements in hemodynamic/anatomical and complete tributary ligation ensured symptom resolution. Patients 4, 11, and 13 achieved imaging success with symptom recurrence (hematuria or proteinuria). Incomplete intraoperative decompression—most notably insufficient tributary division—likely contributed to symptom recurrence.

**Table IV:** Preoperative and postoperative data of patients who achieved only imaging success or symptomatic success

Patient number	Results	Preoperative			Intraoperative dissection of LRV branches/full dissociation of SMA	Postoperative		
		LRV diameter ratio	LRV peak velocity ratio	AM angle		LRV diameter ratio	LRV peak velocity ratio	AM angle
2	Stent migration	4.4	5.9	23	Yes	3.2	1.7	52
4	Proteinuria	6.3	6.1	19	No	1.9	4.7	21
11	Gross hematuria	4.9	6.2	22	No	2.0	4.2	31
13	Gross hematuria	4.2	5.6	21	No	1.7	3.8	25

*AM,* Aortomesenteric; *LRV,* left renal vein; *SMA,* superior mesenteric artery.

**Fig 5:**
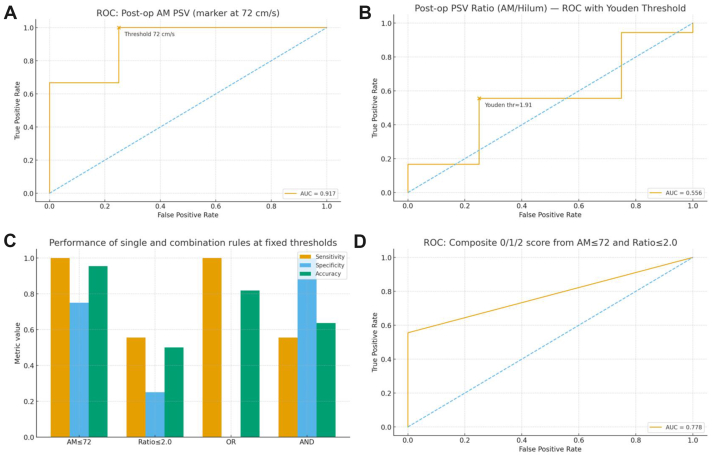
Threshold-based discrimination and combination strategies under the strict complete-success end point (n = 22). **(A)** Receiver operating characteristic (*ROC*) of postoperative aortomesenteric peak systolic velocity (*AM PSV*) of the left renal vein (*LRV*), with the Youden-optimal cutoff indicated on the curve. **(B)** ROC of the postoperative AM/hilum PSV ratio, with the Youden-optimal cutoff indicated. **(C)** Bar chart comparing sensitivity, specificity, and accuracy profiles across four fixed-threshold rules (AM PSV alone; ratio alone; OR rule; AND rule). **(D)** ROC of a composite 0/1/2 score derived from the two thresholds (0 = neither criterion met; 1 = one criterion met; 2 = both criteria met).

Using the strict definition of complete success, the data-driven ROC-Youden analysis identified a postoperative AM LRV PSV of ≤72 cm/s as the primary attainment threshold. At this cutoff, sensitivity was 1.00, specificity 0.75, and accuracy 0.95 (Supplementary Table I, online only); AM PSV as a continuous marker yielded an AUC ≈0.917 (Fig 5, *A*). As a sensitivity/replicability metric, the postoperative AM/hilum PSV ratio showed a Youden-optimal cutoff of ≈1.90 (clinically reported as ≈2.0), with point estimates of sensitivity of ≈0.56, specificity of ≈0.75 (Supplementary Table II, online only), and AUC of ≈0.56 (Fig 5, *B*); bootstrap resampling (4000 iterations) provided 95% confidence intervals for the ratio cutoff, its operating characteristics, and AUC (Supplementary Fig 3, online only). Exploratory combinations of threshold rules showed the expected trade-offs. Using an OR rule—AM PSV ≤72 cm/s or ratio ≤2.0—yielded a sensitivity of 1.00, specificity of 0.00, and accuracy of 0.82. By contrast, the AND rule—AM PSV of ≤72 cm/s and ratio of ≤2.0—increased specificity to 1.00 while reducing sensitivity to 0.56, with an accuracy of 0.64 (Fig 5, *C*; Supplementary Table III, online only); a simple 0/1/2 composite score from these two thresholds achieved an AUC of ≈0.78 (Fig 5, *D*). Importantly, adherence to the intraoperative five-in-a-row process checklist was concordant with achieving the AM-PSV attainment threshold on duplex ultrasound examination, linking procedural standardization to objective postoperative hemodynamic targets and, ultimately, higher complete success rates.

Taken together, our central proposition is to adopt a postoperative AM PSV of ≤72 cm/s as a unified and reproducible primary quantitative end point of attainment, while positioning the ratio of ≈2.0 as a secondary, methodological supplement to support cross-operator and cross-device verification and stratification.

## Discussion

NCS is generally considered a result of extrinsic compression of the LRV between the SMA and AA. LRV compression leads to high venous pressures and impaired drainage, manifesting clinically as hematuria, abdominal pain, and other related symptoms.^13^ The management strategy for NCS primarily depends on symptom severity and disease duration. Nonsurgical management is recommended for patients with mild symptoms and short disease duration. However, surgical intervention is typically required for patients with severe and persistent symptoms.^14^ Our group previously reported successful experience with LEVS for NCS, demonstrating the feasibility and safety of this minimally invasive technique. In the present study, we analyzed 22 surgical cases of NCS and evaluated preoperative and postoperative parameters and critical intraoperative steps. By examining the differences in their outcomes, we identified key factors potentially influencing surgical outcomes, thereby refining the surgical procedures with EVS.

Traditional surgical approaches for NCS included LRV transposition, SMA transposition, open vascular bypass, nephropexy, gonad-vena cava anastomosis, and left renal autotransplantation.^8^ Although these procedures demonstrated efficacy in previous reports, they were associated with significant complications, including anastomotic stenosis or leakage, vascular injury, renal vein thrombosis, retroperitoneal hematoma, and mesenteric ischemia.^15^ Recently, endovascular stenting and EVS emerged as common techniques.^16^ However, endovascular stenting carries inherent risks of complications, including venous thrombosis, stent migration, protrusion into the IVC, and in-stent restenosis, which in severe cases may necessitate left nephrectomy.^11^ Furthermore, patients undergoing endovascular stenting require lifelong or prolonged antiplatelet therapy, increasing their financial burden and potential bleeding risk.^2^^,^^17^ In contrast, EVS represents a more promising therapeutic approach.^18^ This technique eliminates the need for vascular reanastomosis or long-term anticoagulation.^19^ Moreover, the stent material has excellent biocompatibility within the retroperitoneal space. For example, highly stretchable ePTFE tubes are excellent for customizable covered stents with favorable functionality and biocompatibility in stent graft applications.^20^ EVS is suitable for laparoscopic or robotic surgery.^21^ A recent report showed successful outcomes in 13 patients with LEVS, supporting its feasibility and safety profiles.^22^ Similarly, another report showed a high level of safety and efficacy outcomes in six patients with RA-EVS, although within a short follow-up duration.^23^ Despite these successful reports, our clinical experience indicates that a subset of patients still experiences symptom recurrence postoperatively.^12^ Therefore, further accumulation of clinical experience is essential to determine critical factors for a favorable outcome.

In this retrospective cohort of 22 surgical NCS cases, we examined preoperative and postoperative duplex ultrasound parameters alongside critical intraoperative steps and contrasted complete success with suboptimal outcomes. Surgical approach was not randomized and reflected routine clinical practice. Comparative baseline data by approach are provided in Table II and demonstrate several imbalances between the LEVS and RA-EVS groups, suggesting potential selection bias and residual confounding. Accordingly, any approach-stratified interpretation should be considered descriptive and hypothesis generating rather than evidence of equivalence or superiority of one approach over the other. The primary inferences of this study, therefore, focus on the overall cohort, including a reproducible operative process framework and postoperative hemodynamic attainment targets associated with complete success. A distinct fibrotic ring encircling the LRV near its confluence with the IVC was present in 19 of the 22 patients (86.4%) and likely contributed to extrinsic compression; meticulous dissection and removal proved essential. Overall, complete success was achieved in 18 of the 22 patients (81.8%). Failures clustered around two mechanisms: (1) device-related issues (eg, migration) and (2) incomplete hemodynamic decompression, often when tributary division or SMA mobilization was suboptimal. These observations supported a shift from purely anatomical surrogates toward objective hemodynamic attainment on postoperative DUS examination. Using the strict end point of complete success (both symptomatic and imaging success, without recurrence), our data-driven analysis identified a postoperative AM LRV PSV of ≤72 cm/s as the primary attainment threshold. At this cutoff, sensitivity was 1.00, specificity 0.75, and accuracy 0.95; AM PSV as a continuous marker yielded an AUC of ≈0.917 (Fig 5, *A*). As a sensitivity/replicability metric, the postoperative AM/hilum PSV ratio showed a Youden-optimal cutoff of ≈1.90 (clinically reported as ≈2.0) with modest discrimination (AUC ≈0.56) and wide bootstrap uncertainty in this small sample (Fig 5, *B*). Clinically, this means that some patients can achieve strict complete success even if the ratio remains slightly above 1.90, likely reflecting (1) early postoperative variability in hilum velocities, (2) measurement noise, and (3) the time lag between anatomical decompression and full hemodynamic remodeling. Accordingly, we position the ratio as a secondary, methodological supplement for cross-operator/device comparability rather than a standalone gatekeeper.

Exploratory combinations of fixed rules confirmed the expected trade-offs: an OR rule (AM PSV of ≤72 cm/s or ratio of ≤2.0) maximized sensitivity (1.00) but eliminated specificity (0.00; accuracy of 0.82), whereas an AND rule (AM PSV of ≤72 cm/s and ratio of ≤2.0) increased specificity (1.00) while reducing sensitivity (0.56; accuracy of 0.64). A simple 0/1/2 composite score derived from these two thresholds achieved an AUC of ≈0.78 (Fig 5, *C* and *D*). These findings reinforce the primacy of an AM PSV of ≤72 cm/s as a single, actionable target that balances detection and false positives in routine practice. Methodologically, we translated these insights into a process-outcome loop. We standardized EVS around a five-step intraoperative checklist—resection of the fibrotic ring, proper length tailoring/placement to augment LRV caliber, sufficient SMA mobilization to increase the AM angle, complete division of LRV tributaries, and secure anterior fixation of the stent (five-in-a-row). This process measure was concordant with attaining the postoperative DUS target (AM PSV of ≤72 cm/s), linking doing the right steps with meeting the hemodynamic threshold, and ultimately to higher complete success rates. In practice, an AM PSV of ≤72 cm/s serves as the primary quantitative end point for postoperative attainment; the ratio of ≈2.0 provides secondary corroboration and helps to stratify borderline cases and follow-up trajectories across operators and devices.

This study has limitations inherent to its retrospective design. Surgical approach was not assigned randomly, and baseline imbalances between the LEVS and RA-EVS groups indicate potential selection bias and residual confounding. Consequently, between-approach comparisons are exploratory and hypothesis generating only. The rarity of NCS and the availability of conservative options constrain sample size and statistical power, and the small sample size precluded robust adjustment methods such as propensity score matching without compromising statistical stability. The absence of a contemporaneous control group (eg, endovascular stenting) limits comparative effectiveness inference. These postoperative hemodynamic goals may help to predict outcome and standardize postoperative assessment, but they do not provide intraoperative guidance for real-time correction during the procedure. In addition, this series focused on anterior NCS, and the applicability of EVS to other anatomical scenarios remains uncertain; management strategies for a persistently stretched LRV after EVS were not systematically evaluated. Future work should validate the AM PSV of ≤72 cm/s target in larger, multicenter cohorts with standardized DUS protocols, assess learning curve effects of the five-in-a-row, and explore adjunctive imaging or physiologic metrics to refine risk stratification.

LEVS or RA-EVS is a feasible and effective treatment for NCS. We propose a postoperative AM PSV of ≤72 cm/s as a unified, reproducible primary attainment end point, with an AM/hilum ratio of ≈2.0 as a secondary, replicability-oriented metric. Coupling this hemodynamic target with a standardized five-in-a-row operative checklist provides a coherent framework that enhances procedural reproducibility, aligns intraoperative execution with objective postoperative goals, and provides sustained symptom relief during follow-up.

## Conclusions

In this retrospective single-center series, LEVS and RA-EVS for anterior NCS was associated with clinical and imaging improvements over the available follow-up period. A standardized operative process framework and postoperative hemodynamic attainment targets were associated with complete success, supporting a reproducible approach to postoperative assessment. These postoperative goals may help to predict outcomes, but do not provide intraoperative guidance for real-time correction. Given the retrospective design, nonrandomized approach selection, and limited long-term cumulative outcomes, prospective studies with standardized follow-up are needed to validate these findings and define long-term durability.

The authors are grateful for the technical support provided by Dong Wang and Xiang Jin from the Department of Ultrasonography, the Third Central Hospital of Tianjin. We are also thankful to Drs Xinan Jiang, Dengbao Li, and Siwen Zhong from the Department of Urology, the Affiliated Hospital of Guizhou Medical University, for their clinical assistance.

## Author contributions

Conception and design: ST, FMC

Analysis and interpretation: ST, KL, FC, ZL, WW

Data collection: KL, FC, SL, JZ, HS, FMC

Writing the article: ST, FC

Critical revision of the article: ST, KL, SL, ZL, JZ, WW, HS, FMC

Final approval of the article: ST, KL, FC, SL, ZL, JZ, WW, HS, FMC

Statistical analysis: ST, KL, FC, SL, ZL, JZ, WW, HS

Obtained funding: ST, FMC

Overall responsibility: FC

## Funding

This work was partially supported by the Scientific Research Program of the Tianjin Municipal Education Commission (Grant #2024ZX020) to Dr. Shuai Tang.

## Disclosures

None.

## Appendix (online only)

Supplementary Figure 1 Baseline balance by surgical approach. Love plot showing absolute standardized mean differences for preoperative baseline variables comparing laparoscopic extravascular stenting (*LEVS*) and robot-assisted EVS (*RA-EVS*) (Table I). Dashed lines at 0.10 and 0.20 indicate commonly used thresholds for small and moderate imbalance. *Abs SMD*, absolute standardized mean difference; *AM*, aortomesenteric; *PSV*, peak systolic velocity; *SMA*, superior mesenteric artery.

Supplementary Figure 2 One-year postoperative computed tomography (CT) views after extravascular stenting (EVS) for nutcracker syndrome (NCS). **(A, C)** Preoperative compression of the left renal vein (*LRV*) between the abdominal aorta (*AA*) and the superior mesenteric artery (*SMA*). **(B, D)** CT scans at 1 year demonstrating the extravascular stent in place with no evidence of cuff compression, consistent with the standardized 3-month postoperative imaging assessment. *ES*, extravascular stent.

Supplementary Figure 3 Postoperative aortomesenteric (*AM*)/hilum peak systolic velocity (*PSV*) ratio: distribution and model-based probability under the strict complete-success end point (n = 22). **(A)** Distribution of the postoperative AM/hilum PSV ratio stratified by outcome (complete success vs not complete success); the vertical dashed line indicates the Youden cutoff of approximately 1.90 (clinically approximately 2.0). **(B)** Logistic model-predicted probability of complete success as a function of the postoperative AM/hilum PSV ratio, showing a monotonic increase in predicted success as the ratio decreases; the vertical dashed line marks the Youden cutoff of approximately 1.90 (approximately 2.0).
