# Supplementary figures and images for: Laparoscopic and robotic extravascular stenting of the left renal vein for anterior nutcracker syndrome in a single-center series
Source: J Vasc Surg Venous Lymphat Disord. 2026 Feb 12;14(3):102459. doi: 10.1016/j.jvsv.2026.102459 (PMC12992085; doi:10.1016/j.jvsv.2026.102459)

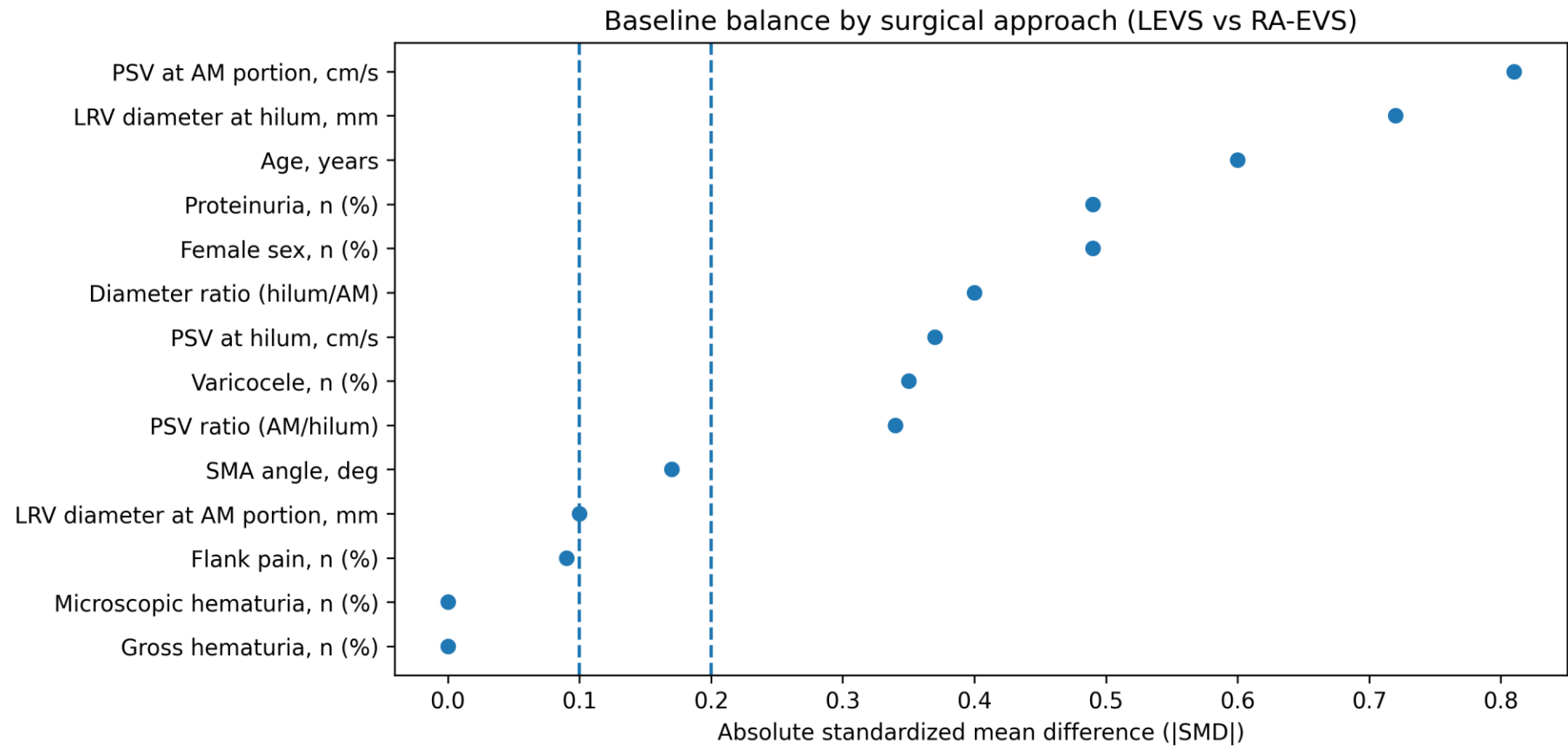

Supplement: Supplementary Figure 1 — Baseline balance by surgical approach. Love plot showing absolute standardized mean differences for preoperative baseline variables comparing laparoscopic extravascular stenting (LEVS) and robot-assisted EVS (RA-EVS) (Table I). Dashed lines at 0.10 and 0.20 indicate commonly used thresholds for small and moderate imbalance. Abs SMD, absolute standardized mean difference; AM, aortomesenteric; PSV, peak systolic velocity; SMA, superior mesenteric artery. [file mmc1.pdf]

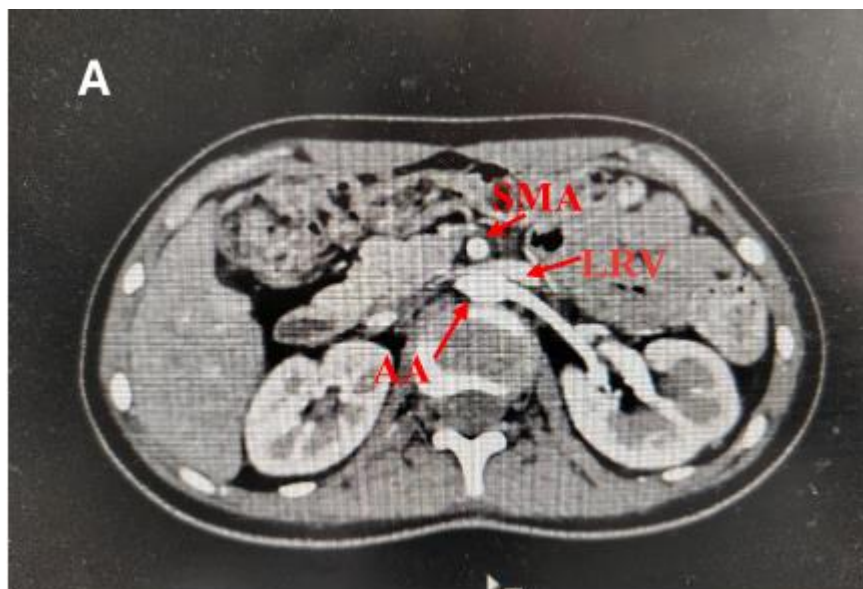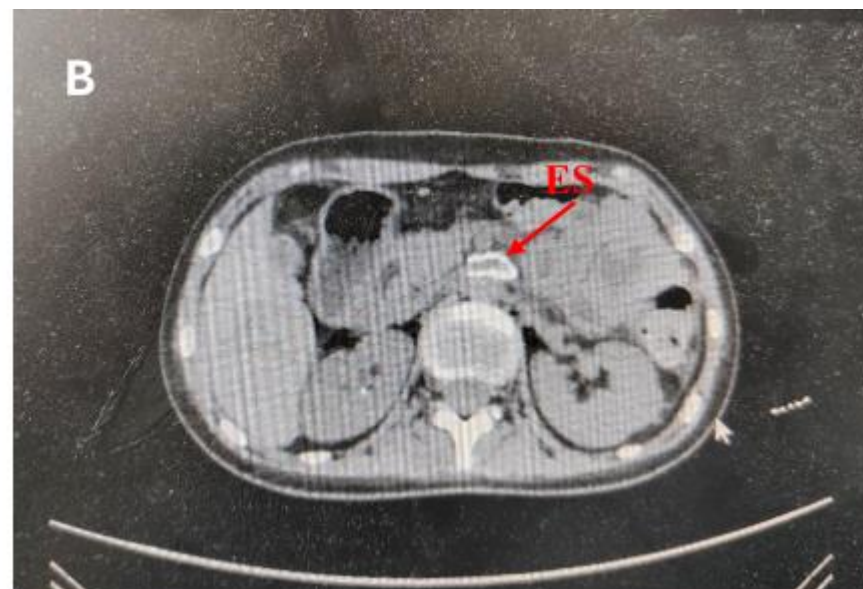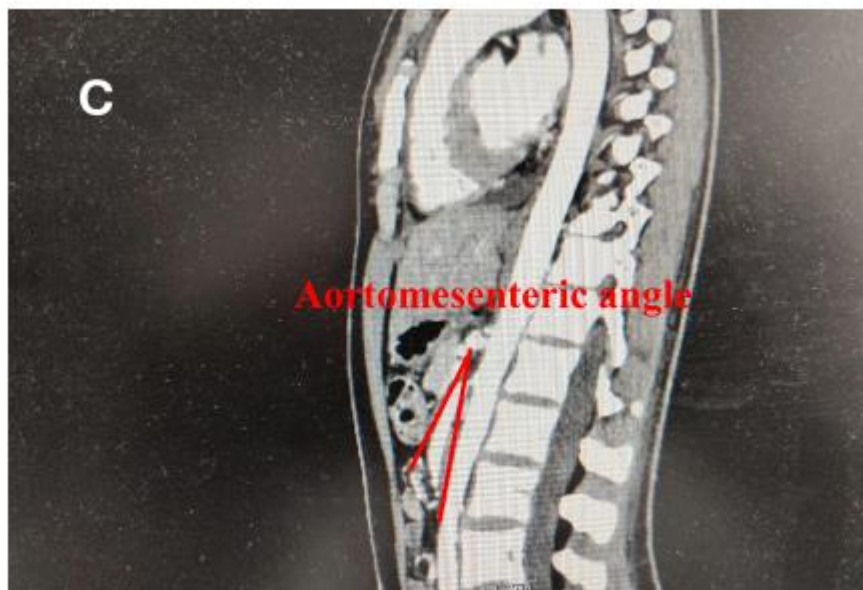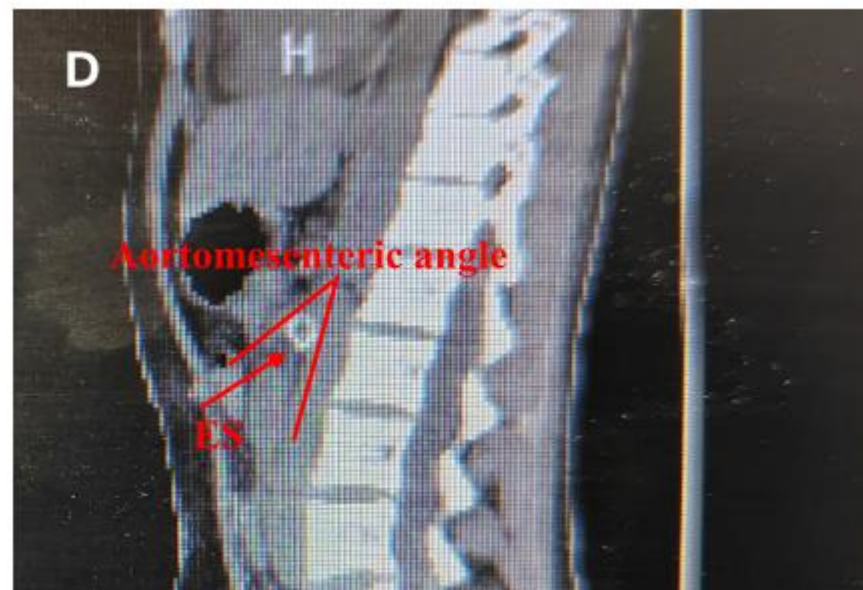

Supplement: Supplementary Figure 2 — One-year postoperative computed tomography (CT) views after extravascular stenting (EVS) for nutcracker syndrome (NCS). (A, C) Preoperative compression of the left renal vein (LRV) between the abdominal aorta (AA) and the superior mesenteric artery (SMA). (B, D) CT scans at 1 year demonstrating the extravascular stent in place with no evidence of cuff compression, consistent with the standardized 3-month postoperative imaging assessment. ES, extravascular stent. [file mmc2.pdf]

**A**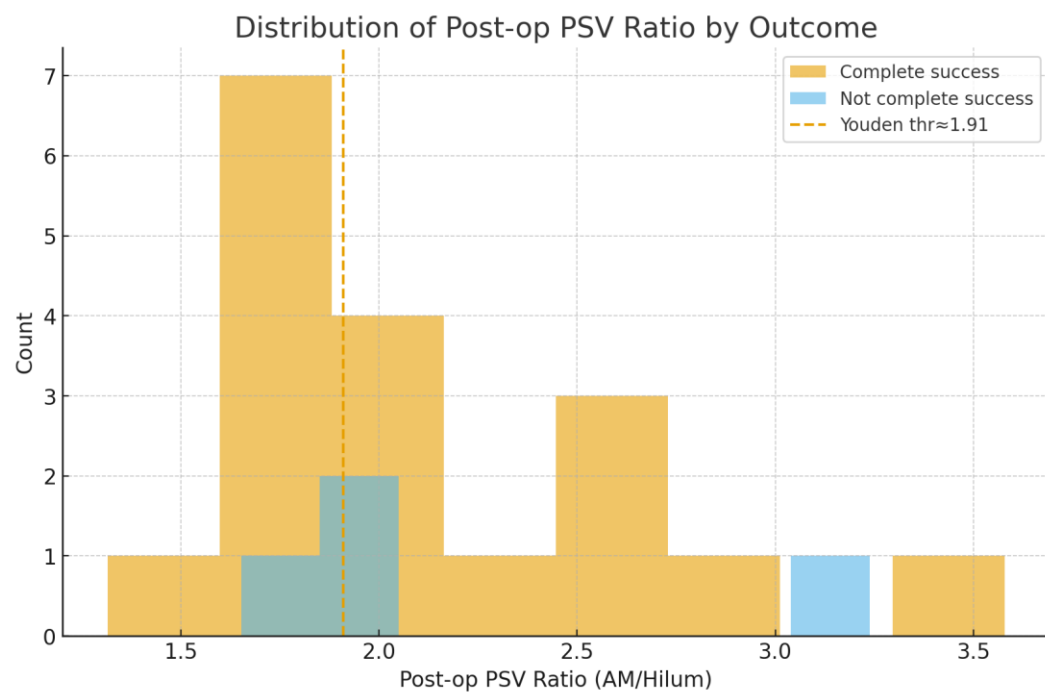**B**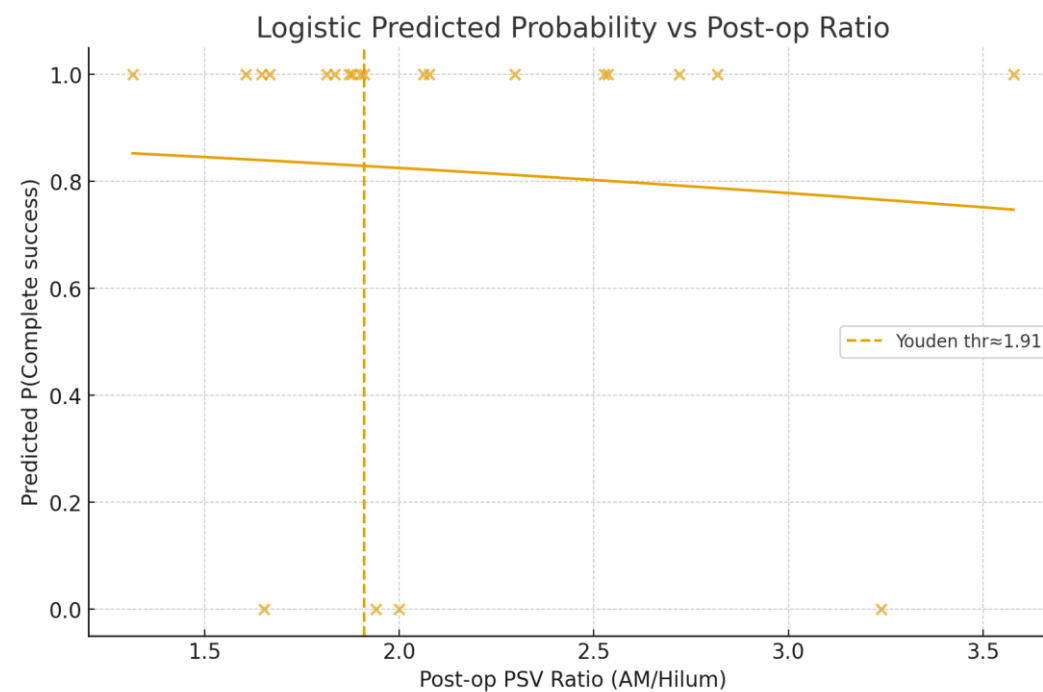

Supplement: Supplementary Figure 3 — Postoperative aortomesenteric (AM)/hilum peak systolic velocity (PSV) ratio: distribution and model-based probability under the strict complete-success end point (n = 22). (A) Distribution of the postoperative AM/hilum PSV ratio stratified by outcome (complete success vs not complete success); the vertical dashed line indicates the Youden cutoff of approximately 1.90 (clinically approximately 2.0). (B) Logistic model-predicted probability of complete success as a function of the postoperative AM/hilum PSV ratio, showing a monotonic increase in predicted success as the ratio decreases; the vertical dashed line marks the Youden cutoff of approximately 1.90 (approximately 2.0). [file mmc3.pdf]
