# Supplementary material for: Laparoscopic and robotic extravascular stenting of the left renal vein for anterior nutcracker syndrome in a single-center series
Source: J Vasc Surg Venous Lymphat Disord. 2026 Feb 12;14(3):102459. doi: 10.1016/j.jvsv.2026.102459 (PMC12992085; doi:10.1016/j.jvsv.2026.102459)
Supplement: Supplementary Tables [file mmc4.docx]

**Supplementary Table 1. Diagnostic performance of post-operative aortomesenteric LRV PSV at the fixed threshold of ≤72 cm/s for the strict complete-success endpoint.**

| Predictor | Threshold | N | TP | TN | FP | FN | Sensitivity | Specificity | Accuracy | PPV | NPV | AUC (continuous) |
| --- | --- | --- | --- | --- | --- | --- | --- | --- | --- | --- | --- | --- |
| Post-op AM PSV (cm/s) | ≤ 72 | 22 | 18 | 3 | 1 | 0 | 1.0 | 0.75 | 0.95454 | 0.94736 | 1.0 | 0.91666 |

Abbreviations: LRV, left renal vein; AM, aortomesenteric; PSV, peak systolic velocity; PPV, positive predictive value; NPV, negative predictive value; AUC, area under the ROC curve.

**Supplementary Table 2. Bootstrap (4,000 iterations) summary for the post-operative AM/Hilum PSV ratio: Youden-optimal cutoff and operating characteristics with 95% CIs.**

| Metric | Point | Boot 95%CI low | Boot 95%CI high |
| --- | --- | --- | --- |
| Youden optimal threshold (ratio) | 1.90909 | 1.60606 | 2.81818 |
| AUC (ratio) | 0.55556 | 0.19048 | 0.90777 |
| Sensitivity @Youden | 0.55556 | 0.09524 | 1.00000 |
| Specificity @Youden | 0.75000 | 0.25000 | 1.00000 |
| Accuracy @Youden | 0.59091 | 0.18182 | 0.95455 |
| PPV @Youden | 0.90909 | 0.77778 | 1.00000 |
| NPV @Youden | 0.27273 | 0.09091 | 1.00000 |

Abbreviations: PPV, positive predictive value; NPV, negative predictive value; AUC, area under the ROC curve; CI, confidence interval.

**Supplementary Table 3. Operating characteristics of single and combination threshold rules with confusion-matrix counts.**

| Rule | Sensitivity | Specificity | Accuracy | TP | TN | FP | FN | AUC/Score |
| --- | --- | --- | --- | --- | --- | --- | --- | --- |
| AM PSV ≤72 (single) | 1.0 | 0.75 | 0.95455 | 18 | 3 | 1 | 0 |  |
| Ratio ≤2.0 (single) | 0.55556 | 0.25 | 0.5 | 10 | 1 | 3 | 8 |  |
| OR (≤72 OR ≤2.0) | 1.0 | 0.0 | 0.81818 | 18 | 0 | 4 | 0 | 0.77778 |
| AND (≤72 AND ≤2.0) | 0.55556 | 1.0 | 0.63636 | 10 | 4 | 0 | 8 | 0.77778 |

Abbreviations: TP, true positive; TN, true negative; FP, false positive; FN, false negative; AM, aortomesenteric; PSV, peak systolic velocity; AUC, area under the ROC curve.
